# Comment on “Intestinal Helminths in Different Species of Rodents in North Khorasan Province, Northeast of Iran”

**Published:** 2019

**Authors:** Hassan BORJI

**Affiliations:** Department of Pathobiology, School of Veterinary Medicine, Ferdowsi University of Mashhad, Mashhad, Iran

## Dear Editor-in-Chief

I read with a great interest the paper by Arzamani et al. entitled” Intestinal Helminths in Different Species of Rodents in North Khorasan Province, Northeast of Iran” recently published in Iranian Journal of Parasitology (1).

Arzamani et al. have been reported *Taenia taeniaeformis* from the rodents surprised me. *T. taeniaeformis* is a parasitic tapeworm with worldwide distribution, with the adult found in the cat as the primary definitive hosts and the rodents as the intermediate hosts. This is the only species of *Taenia* typically reported from the domestic cats around the world. Sometime dogs can also be the definitive hosts. The intermediate hosts of this tape-worm are primarily rodents and less frequently lagomorphs (rabbits) which segmented larval stage found in the liver of rodents.

The larval stage was first described as *Cysticercus fasciolaris*. The genus, Hydatigera, was famous for this larval form. In the effort to division the genus *Taenia* on the basis of different life cycle patterns, the species in the cat, *T. taeniaeformis*, was allocated to the genus Hydatigera. For the first time, Küchenmeister (2) was shown that the larval stage in the rodents could infect cats, and Leuckart (3) completed the whole life cycle and infection of rodents with eggs and infection of cats with the larval stage in the laboratory.

Other points and mistakes in this paper are as follows:

1- They demonstrated that “Thirteen species of helminths parasites were found in 13 species of rodents” not correlated with table 1 (1). In this table, 12 species of rodents was mentioned.
2- In material and methods noted that “Their alimentary canals were removed in the laboratory and the parasites were isolated from rodent intestines”. However, in result, *Cysticercus fasciolaris* was reported from the rodents which are develops mainly in the liver of rodents, occasionally in the abdominal wall and kidney. Moreover, lesions of *C. fasciolaris* in the liver of rodents have also been shown in figure 3 of that article (1).
3- In results indicated that S. *obvelata* and *A. tetraptera* were the most frequent parasites and *C. fasciolaris* were found only in one rodent which is in contrast with data presented in Table 2. In this table, *C. fasciolaris* was reported as the most frequent parasites. Of course, I believe that high frequency of *C. fasciolaris* from rodents is uncommon and unreal based on previous studies (4, 5).
4- In figure 3 (1), cysts of *C. fasciolaris* in liver was showed that is not informative. It would be valuable to show some pictures from hooks and scolex of larvae for characterization of cyst (5).
5- Frequency of infected rodents presented in table 2 (1) do not address the key question on abundance, mean intensity, and range of helminth species in these rodents.
6- In the result, the authors stated that “*S. obvelata* were found in one *Ellobius fuscocapillus*” which is not in agreement with information existing in table 2 (1). In this table, only *C. fasciolaris* was collected from *Ellobius fuscocapillus* and *S. obvelata* was collected from *Meriones persicus.*
7- In discussion, the authors indicated that “Considering the variation of parasites, eight species of helminth were found in *M. persicus”* which is in difference with data presented in table 2 (1). In this table, nine species of helminth were found in *M. persicus.* Moreover, in the next section of discussion, the authors indicated that” *T. muris* and *S. obvelata* were observed in *Apodemus witherbyi* for the first time in Iran” which is not in consistent with table 2 (1). In this table, *T. muris* and *S. obvelata* was collected from *Meriones persicus*
8- In Acknowledgements, the authors declared that “Voucher specimens were stored at Ferdowsi University and Vector-borne Diseases Research Center Museum of North Khorasan University of Medical Sciences, Iran”. Nevertheless, it is needed to deposit all of parasites collected from the rodents for the first time in Iranian National Parasitology Museum (INPM) with a code number for preserving their genetic resources and to be used in research and education.
